# Mesenchymal stromal cells support endothelial cell interactions in an intramuscular islet transplantation model

**DOI:** 10.1186/s40340-015-0010-9

**Published:** 2015-09-30

**Authors:** Moa Fransson, Johan Brännström, Ida Duprez, Magnus Essand, Katarina Le Blanc, Olle Korsgren, Peetra U. Magnusson

**Affiliations:** Department of Immunology, Genetics and Pathology, Division of Clinical Immunology, Uppsala, Sweden; Department of Clinical Immunology and Transfusion Medicine, Karolinska Institutet and Hematology Center at Karolinska University Hospital, Huddinge, Sweden; Department of Immunology, Genetics and Pathology, The Rudbeck Laboratory, Dag Hammarskjölds Väg 20, SE-751 85 Uppsala, Sweden

**Keywords:** Mesenchymal stromal cell, Islets of Langerhans, Transplantation, Endothelial cells

## Abstract

**Background:**

Mesenchymal stromal cells (MSC) have been under investigation for a number of therapies and have lately been in focus as immunosuppressive actors in the field of transplantation. Herein we have extended our previously published *in vitro* model of MSC-islets in an experimental setting of islet transplantation to the abdominal muscle.

Human islets coated with luciferase-GFP transduced human MSC were transplanted to the abdomen muscle tissue of NOD-scid ILR2γ^null^ mice and cellular interactions were investigated by confocal microscopy.

**Results:**

The MSC reduced fibrotic encapsulation and facilitated endothelial cell interactions. In particular, we show a decreased fraction of αSMA expressing fibrotic tissue surrounding the graft in presence of MSC-islets compared to islets solely distributed into the muscle tissue. Also, in the presence of MSC, human islet endothelial cells migrated from the center of the graft out into the surrounding tissue forming chimeric blood vessels with recipient endothelial cells. Further, in the graft periphery, MSC were seen interacting with infiltrating macrophages.

**Conclusions:**

Here, in our experimental *in vivo* model of composite human islets and luciferase-GFP-transduced human MSC, we enable the visualization of close interactions between the MSC and the surrounding tissue. In this model of transplantation the MSC contribute to reduced fibrosis and increased islet endothelial cell migration. Furthermore, the MSC interact with the recipient vasculature and infiltrating macrophages.

**Electronic supplementary material:**

The online version of this article (doi:10.1186/s40340-015-0010-9) contains supplementary material, which is available to authorized users.

## Background

Mesenchymal stromal cells (MSC) are a subpopulation of multipotent cells originally identified in the bone marrow [1]. MSC are characterized by their fibroblast-like appearance, differentiation and colony forming unit capacity including their rapid adherence to plastic surfaces [2]. MSC have been used in both experimental models and in the clinical setting as immunosuppressive treatment [3, 4] and catalyzers of endothelial cell sprout formation [5]. The *in vitro* immunosuppressive capacity combined with proven therapeutic efficacy has paved the way of MSC in the clinic. MSC in an allogeneic nonhuman primate model of islet transplantation showed increased engraftment, indicating a capacity for these cells to reduce rejection [6]. Safety concerns and efficacy of MSC in solid organ transplantation are currently under investigation but so far they have proven to be safe and so far no detrimental effects have been reported [7]. MSC have further been under investigation in a clinical trial as immune modulatory therapy for diabetic patients where early onset type 1 diabetic patients received autologous MSC in an attempt to halt the disease (ClinicalTrials.gov Identifier: NTC01068951 [8]).

MSC have also been under investigation in the transplantation setting of islets (ClinicalTrials.gov Identifier: NCT01967186). Today islets are transplanted to the portal vein of the liver. Unfortunately, due to the instant blood mediated inflammatory reaction (IBMIR) a substantial fraction of islets are destroyed and multiple infusions of islets are usually needed to acquire insulin independence [9]. Therefore, alternative transplantation sites such as the striated muscle have been investigated [10]. The muscle as a transplantation site has shown great potential to support islet revascularization in *in vivo* experimental models [11]. MSC could facilitate the engraftment processes both as immune regulators but also as supporters for the ingrowth of recipient’s vasculature and by producers of stimulatory growth factors [12]. In our previous *in vitro* studies we have shown that the presence of MSC contributed to increase sprout formation of endothelial cells into fibrin gels after being coated onto islets [13]. One benefit of creating composite islets i.e. coating the MSC onto the islet surface instead of performing co-transplantation of MSC in suspension with islets is besides increased possibility of cellular interactions, also a greater possibility of the MSC to reside during a prolonged time at the site of transplantation.

Herein, we present an *in vivo* normoglycemic experimental model of islet transplantation utilizing human composite MSC-islets. MSC expressed GFP/luciferase to enable *in vivo* imaging studies over time and *ex vivo* confocal analysis post explantation. The MSC-islets were transplanted to the abdominal muscle of NOD-scid ILR2γ^null^ mice to improve the engraftment of human cells [14] and analyzed three days to seven days post transplantation for revascularization, infiltration and fibrosis. Our results provide knowledge about the close interactions between the MSC, the recipient’s vasculature and the endogenous islet endothelial cells as well as the accumulation of macrophages.

## Results

### Detection of luciferase/GFP-transduced MSC after transplantation

**Fig. 1:**
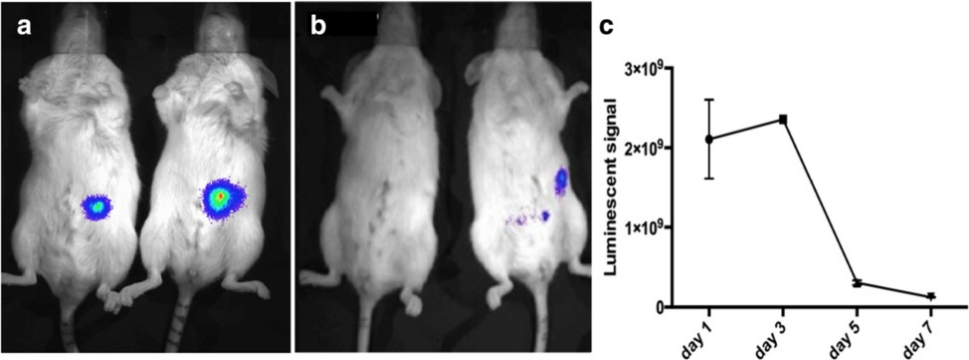
Transplantation of luciferase^+^GFP^+^ MSC-islets into NOD-scid ILR2γ^null^ mice. (**a**) Luciferace signal in MSC-islet mice 3 days post transplantation and (**b**) 6 days post transplantation. **c** Quantification of luciferase signal from the day of transplantation until 7 days post transplantation.

Herein, to create composite islets GFP/luciferase-expressing MSC were coated onto the islets before transplantation to the abdominal muscle. Images of control and MSC-islet grafts after injection into the muscle tissues showed similar deposits of islets (Additional file 1: Figure S1). Three days post transplantation the luciferase expression in the MSC was clearly visualized as shown in Fig. 1a. One-week post transplantation, however, the luciferase expression was reduced (Fig. 1b). Analyzes of the luciferase signal showed that approximately 80–90 % of the initial signal was lost upon day 7 (Fig. 1c). One day post transplantation the luciferase signal in the animals were more or less gathered in a localized spot as shown in Fig. 1a. At the same time point a second luciferase spot at distance from the major graft signal was detected in three animals (Additional file 2: Figure S2).

### Analysis of the islet graft size

**Fig. 2:**
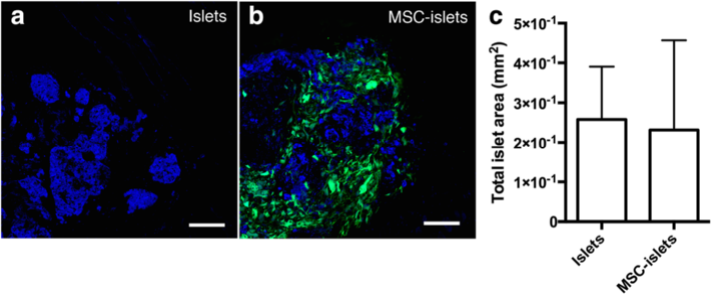
Analysis of the islet graft size. **a** Control islet (blue) graft in the muscle tissue on day 7 post transplantation in comparison to (**b**) MSC-islet graft, where the islets (blue) are surrounded by MSC (green). **c** Quantification of the islet mass in the graft area of control islets and MSC-islets. Bars = 100 um

To investigate the islet mass in the two groups image quantification of the graft area of sectioned tissues was performed. Seven days post transplantation the islet graft of the longitudinal sectioned muscle tissue was investigated in control animals (Fig. 2a, islets in blue) and in the MSC-islet graft (Fig. 2b, green MSC and islets in blue). Quantification of the total islet area in the analyzed sections was similar within the two groups (Fig. 2c). Additional images of the sectioned islet and MSC-islet grafts at day 7 are shown in Additional file 3: Figure S3.

### MSC act as an interactive barrier against accumulating macrophages

**Fig. 3:**
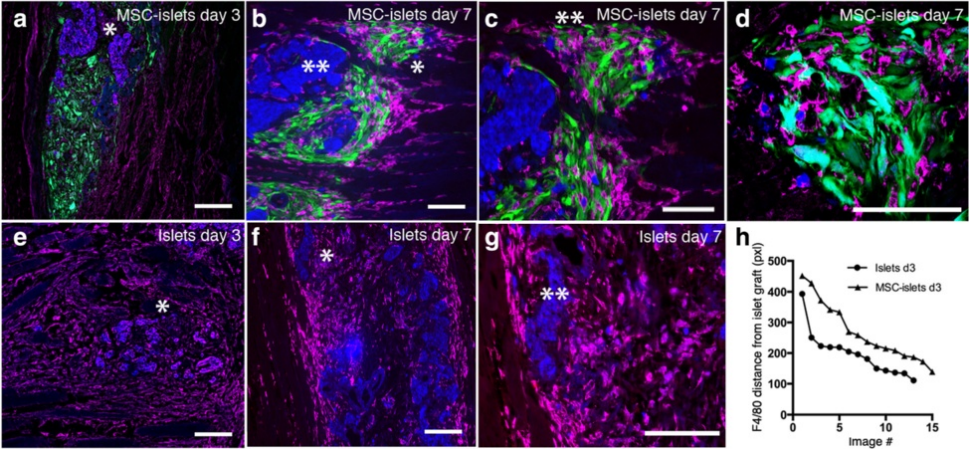
Accumulation of inflammatory cells within the graft area. **a** The MSC (green) intermingled with F4/80^+^ expressing macrophages (purple) close to the islet mass (blue,*) at 3 days post transplantation. **b** MSC interacting (green,*) with F4/80^+^ macrophages (purple) at distance from the islet graft (blue,**) at 7 days post transplantation. **c** Increased magnification of Fig. 2b showing the interactions between F4/80^+^ expressing macrophages and MSC. **d** Close up of the MSC-F4/80 region in Fig. 3c shows MSC intermingling with the macrophages. **e** Infiltrating F4/80^+^ expressing macrophages (purple) into control islet mass (blue,*) 3 days post transplantation. **f** The infiltration of F4/80^+^ macrophages (*) into the islet graft increased even further upon day 7. **g** Increased magnification of the infiltrated area in Fig. 3f. **h** Distance of F4/80 positive cells relative to the islet graft in MSC-islets (n = 4, black triangles) compared to control islet grafts (n = 3, black circles) in analyzed images of the investigated animals. Bars = 100 um

In the presence of MSC three days post transplantation, the accumulating F4/80^+^ macrophages steered into interactions with the MSC surrounding the islet graft (Fig. 3a, MSC in green interacting with F4/80^+^ cells in purple) while in the group only receiving islets, accumulation of macrophages was observed to a large extent around the graft area (Fig. 3e, infiltrating F4/80^+^ cells in purple). In the MSC-islets at one-week post transplantation, infiltrating cells were detected in the MSC area i.e. the periphery of the islet graft (Fig. 3b, *) and only to a small extent in the islet area of the graft (Fig. 3b, **). Close interactions between F4/80^+^ macrophages (purple) and MSC (green) at the periphery of the islet graft (blue) could be observed at this time point (Fig. 3c, **). As visualized in Fig. 3d, macrophages seem to be tightly intermingled with the MSC. While in the control islets upon day 7, infiltrating macrophages were allowed to invade the graft area (Fig. 3f, *) and cluster close to the grafted islet cells (Fig. 3g, increased magnification of asterisk marked region in Fig. 3f). Analysis of the distance of F4/80 expressing cells relative to the islet grafts showed a tendency of accumulating F4/80^+^ cells at a longer distance from MSC-islet grafts compared to control grafts in analyzed images (Fig. 3h).

### Fibrotic process surrounding islets in the absence of MSC

**Fig. 4:**
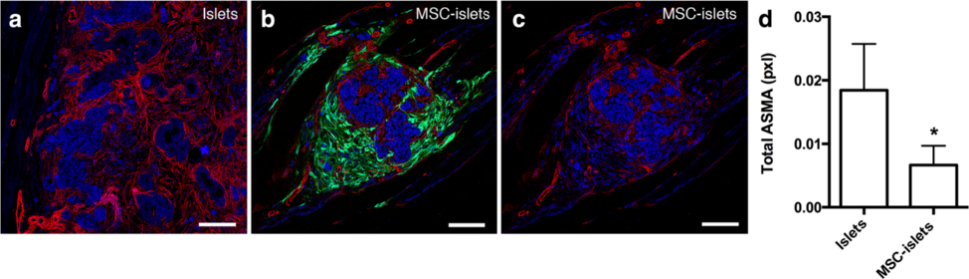
Fibrotic encapsulation of the grafted islets visualized by αSMA expressing tissue one week post transplantation. **a** Control section with a large portion of αSMA^+^ (red) fibrotic tissue surrounding the islets (blue). **b** In the presence of MSC (green) one-week post transplantation only a moderate fibrotic encapsulation (αSMA, red) could be seen surrounding the islets (blue). **c** Tissue in panel B without imaged MSC. **d** Quantification showed significantly lower levels (*p < 0.05) of fibrotic tissue surrounding the graft at 7 days post transplantation in the presence of MSC as measured by total expression of αSMA. Bars = 100 um

Islets transplanted to the muscle tissue without MSC exhibited an active fibrotic process visualized by increased, however not significant, expression of alpha smooth muscle actin (αSMA) three days post transplantation (0.017 ± 0.01 for islets and 0.016 ± 0.004 for MSC-islets, ns (*p* = 0.571)) followed by near to total encapsulation in fibrotic tissue of control islets one week post transplantation (Fig. 4a). In the presence of MSC (green), the amount of αSMA positive stromal tissue (red) surrounding the islet graft was evidently less (Fig.4b & c) compared to islets directly exposed to the muscle tissue (Fig. 4a). Quantification showed significantly lower levels of αSMA positive stromal fibroblastic cells surrounding the MSC-islets compared to control islets one-week post transplantation (Fig. 4d). Assessing degradation of the graft at three days post transplantation by quantifying apoptotic events by the expression of positive apoptotic nuclei stained by ApopTag shown in Additional file 4: Figure S4 revealed similar presence of apoptotic nuclei in control islets compared to MSC-islets (Additional file 4: Figure S4A and B, respectively). Image quantification showed no significant difference between the two groups (Additional file 4: Figure S4C).

### Human islet endothelial cell movement *in vitro* and at site of transplantation

**Fig. 5:**
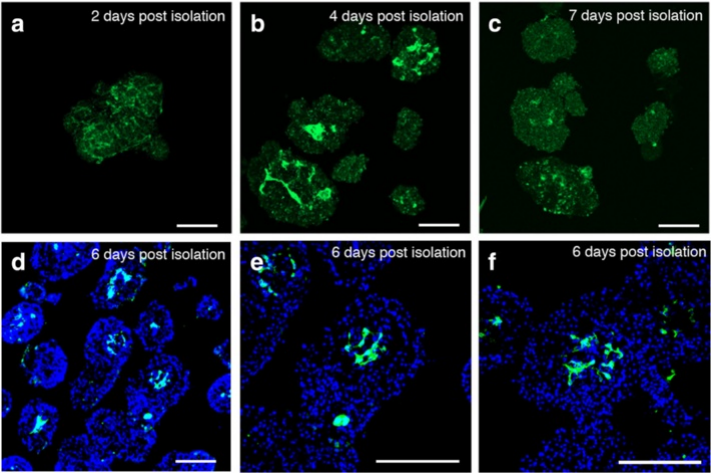
Islet endothelial cell migration in cultured human islets in the absence of MSC. **a** *In situ* CD31 expression (green) two days after isolation showing vessels that are evenly spread throughout the islet. **b** After four days in culture after isolation, the vessels are withdrawn towards the center of the islet and after (**c**) one week in culture, the vessels are loosing their expression of CD31. **d**-**f** Sectioned human islets stained for CD31 (green) at 6 days after isolation. Nuclei stained by DAPI in blue. Bars = 100 um

**Fig. 6:**
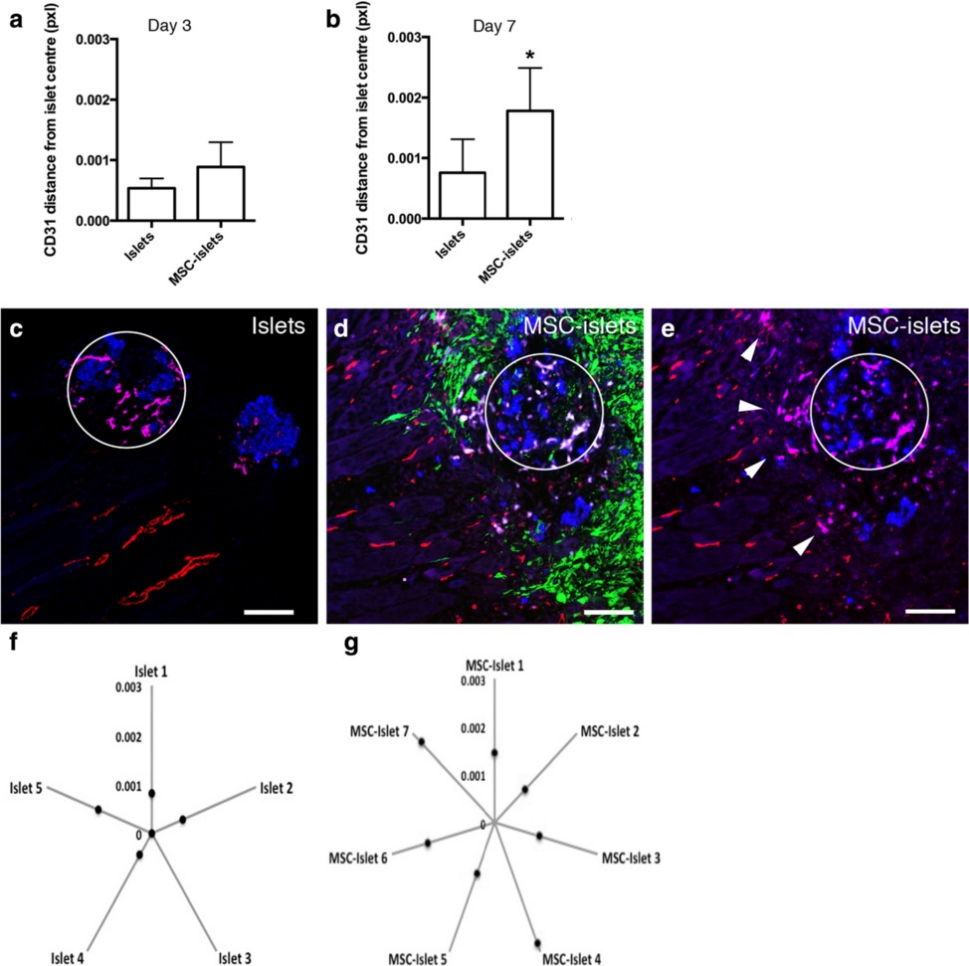
Facilitated islet endothelial cell migration out into the surrounding tissue in the presence of MSC. **a** Mean distance of human CD31 expressing vessels from the center of the islet in control islets and MSC-islets at three days post transplantation (*n* = 4). **b** Mean distance of human CD31 expressing vessels from the center of the islet in control islets compared to MSC-islets at seven days post transplantation (*p < 0.05, islets *n* = 5, MSC-islets *n* = 7) using a Mann–Whitney test. **c** In control sections after one week, the human islet endothelial cells (purple) remained inside the islet graft (blue, centrally marked by a white circle) avoiding direct contact with the recipient vasculature (red). **d** In the MSC-islets (green and blue, respectively, centrally marked by a white circle) the presence of human islet endothelial cells (white/purple) were more prominent at distance of the islet graft at the same time point. **e** Same figure as in panel D excluding the MSC signal in green showing the human islet endothelial cells (purple, marked with white arrow heads) migrating out to the surrounding tissue (day 7). **f** Mean distance of human endothelial cell migration from the center of the control islet grafts in each study object 7 days post transplantation (*n* = 5). **g** Mean distance of human endothelial cell migration from the center of the MSC-islet grafts in each study object 7 days post transplantation (*n* = 7). Bars = 100 um

Investigating the movement of islet endothelial cells over time of *in vitro* cultured human islets showed that the islet endothelial cells were found in the center of the islets (Fig. 5). Human islets were kept in culture for 2 days (Fig. 5a), 4 days (Fig. 5b) and 7 days (Fig. 5c) after isolation and analyzed *in situ* for CD31 expression. The resident endothelial cells were retracted to the centric part of the islet and as the *in vitro* culture progressed the CD31 expression slowly disappeared as shown by small CD31^+^ structures remaining while the autofluorescence of the islets increased. Furthermore, sections of cultured human islets clearly detected CD31^+^ endothelial structures within the islet core upon day 6 of *in vitro* cultures (Fig. 5d-f). A similar scenario of islet endothelial cells localized centrally as the *in vitro* setting of cultured islets was shown *in vivo* where the CD31 expression in control islet grafts was evaluated on day 3 (Fig. 6a) and on day 7 showing human endothelial cells significantly closer to the center of the islet mass at one week post transplantation compared to MSC-islets (Fig. 6b, *p* < 0.05).

### MSC facilitate endogenous and recipient vascular interactions

**Fig. 7:**
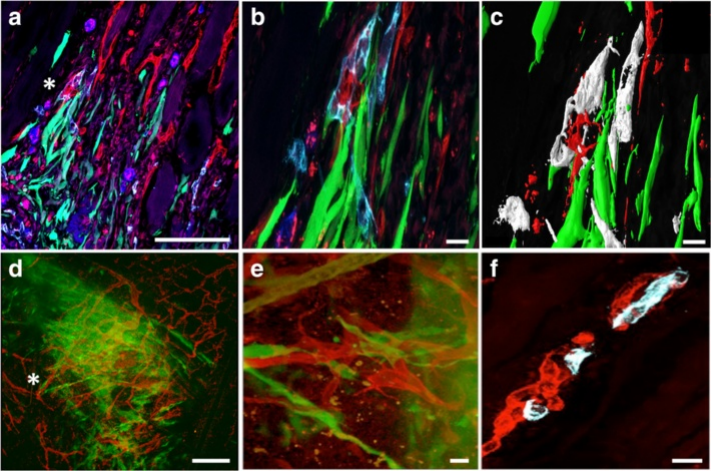
Cellular interactions between MSC and human islet endothelial cells with the recipient vasculature one week post transplantation. **a** Interactions between MSC (green) and the CD31 expressing human islet endothelial cells (light blue) with the recipient vasculature (red). **b** Increased magnification in asterisk marked area of panel A showing the interactions between the MSC and human islet endothelial cells where the MSC are wrapped around the mouse vasculature. **c** Imaris visualization of the image shown in panel b. **d** *In situ* staining and *in situ* confocal microscopy created an overview of the graft area showing sprouting MSC (green) close to the mouse vasculature (red). **e** Increased magnification of the asterisk area in panel d showed MSC in green wrapped around the mouse vasculature in red. **f** Formation of a chimeric blood vessel in the close proximity of the MSC. The human islet endothelial cells (white) are fused with the murine blood vessel (red). Bars = 100 um in panel a and d, 5 um in panel b and c and 10 um in panel e and f

In the control sections, the islet vasculature showed quite dense structures inside the islet area (blue, centrally marked by a white circle) and there were no signs of interactions with the recipient vasculature or the surrounding tissue (Fig. 6c, purple CD31 structures inside the white circle and recipient's red CD31 vessel structures in the periphery). CD31 positive human islet endothelial cells (white/purple) of MSC-islets (green and blue respectively, centrally marked by a white circle) were sprouting further out from the center of the islet compared to controls (Fig. 6d and e, purple structures inside the white circle but also outside marked with arrow heads). The calculated mean distance of endothelial cells migrating from the center of the islet graft was increased in MSC-islets (Fig. 6g) compared to control islets (Fig. 6f) 7 days post transplantation. However, in neither of the groups, a complete revascularization of the graft could be seen at this time point as evaluated by staining for CD31. In the presence of MSC the human islet vasculature was intermingling with the recipient vasculature and at some locations closely as seen in Fig. 7a showing human CD31 in light blue, mouse CD31 in red and MSC in green 7 days after transplantation. The asterisk in panel A indicates the region that is shown in increased magnification in 7B. The interaction of human islet endothelial cells (white) with the recipient vasculature (red) was further envisaged by processing the Fig. 7b with Imaris data visualization (Fig. 7c). Further, *in situ* staining of grafts one-week post transplantation revealed an interactive network between MSC (green) and the recipient vasculature (Fig. 7d, red and 7e showing increased magnification of asterisk marked area in panel d) and chimeric vessel formation (Fig. 7f) of human islet endothelial cells (white) with the recipient vasculature (red).

## Discussion

In the current study we have been able to investigate the interactions between human MSC and human islets in an *in vivo* model of transplantation to enlighten initial cellular events by utilizing advanced imaging techniques. The findings here correlate with our previous *in vitro* model of MSC-islets where it was shown that the presence of MSC induced endothelial cell migration [5] and as an additional suggested mechanism in the *in vivo* setting, the MSC was shown to interact with infiltrating macrophages. Furthermore, the MSC contributed to significantly reduced fibrosis compared to control islets.

Today, clinical islet transplantation suffers from loss of islets in part caused by the IBMIR occurring upon infusion into the portal vein of the liver. Over the past decade there has been an increased interest in alternative transplantation sites due to mentioned obstacles and deterioration of islet graft function post intraportal transplantation. Further, the need of rapid revascularization processes increases the demand on the transplantation site. The striated muscle has a natural ongoing angiogenesis and a high oxygen tension and might therefore to be considered as a preferable site for transplantation [15]. The muscle as a transplantation site has previously shown great potential to support islet revascularization in experimental models of islets transplanted to the immune privileged cremaster muscle [11].

Macrophages and neutrophils are known to infiltrate grafted tissues and cause degradation and tissue damage [16] but also to support revascularization [17]. In our experimental model with transplanted human MSC-islets, we show that the framework of MSC surrounding the islets was interacting with accumulating macrophages. The interactions at depth showed that the MSC seemed to be wrapped around and at some locations fused with the macrophages. Image analysis verified a potential effect by the MSC on day 3 post transplantation as shown by accumulation of F4/80 at a longer distance from the islet graft in the presence of MSC compared to control islets.

One element of consideration within this study is the disappearance of MSC from the transplantation site. Upon clinical intravenous administration of MSC, the cells are trapped within the lung and are thereafter rapidly disappearing. However, as the therapy has effect in situations such as graft versus host disease, there may be a small part of the cells that survive and utilize their function, such as homing to sites of injury or releasing immune modulatory factors [18, 19]. With our data we can confirm that there is a loss of a majority of the transplanted MSC at site of transplantation. By estimation of the luciferase signal the transplanted MSC were reduced by approximately 90 % one-week post transplantation. Still, the remaining MSC were highly active and interacted with the infiltrating cells and migrated out to the surrounding muscle tissue. Others have also shown that MSC have a tendency to disappear from the site of transplantation [20] and that MSC migration from the injured site is dose and time dependent and before all, exclusively CXCR4 dependent [21]. Further, MSC seem to have a systemic effect even when not present at the site [22] so the amount of cells and for how long time the MSC are needed at one particular site is inconclusive. Along with the continuous reduction of the luciferase signal we did not observe any specific pattern of migration. However, there is always a possibility that some MSC detach from the islets upon injection and they can probably be drained from the site of transplantation when the surrounding tissue soak up injected excess liquid. Furthermore, the loss of MSC could of course also be due to cell death. We can only speculate about this, but the luciferase dots that appeared in three of the animals (Additional file 2: Figure S2) may indicate deposit of MSC within the spleen or the liver. 

Pancreatic islets have a rich vascular supply in the native pancreas and some report that islets receive 5–10 % of pancreatic blood flow despite coverage of 1–2 % in the pancreas [23, 24]. Even though we could not see a full revascularization process of the implanted grafts in our transplantation model, we could observe that when the MSC were present, the distance of CD31^+^ islet sprouting endothelial cells were significantly longer than in islets solely distributed into the muscle tissue. In our *in vitro* studies of cultured human islets we have seen that the resident endothelial cells are retracted to the center part of the islet and as the *in vitro* culture progressed, the CD31 expression declined as a reflection of lost CD31 expression from the islet vasculature or actual endothelial cell loss. This can be related to the work of Nyqvist and colleagues [25] where they investigated the expression of CD31 in cultured mouse islets. They further showed a retraction and a loss of islet endothelial cells as early as 3–4 days post isolation. To compare, with our results it was shown that human islet endothelial cells remain within the islet center after 6 days in culture. A phenomenon likely to occur due to increased expression of vascular endothelial growth factor (VEGF) within the hypoxic islet core [26]. MSC are known to produce VEGF [27] and it is not unlikely that growth factors produced by the MSC is a cause of islet endothelial recruitment into the surrounding tissue. MSC have further been shown to produce several trophic molecules that trigger angiogenesis in a model of islet transplantation [28]. If our finding of recruitment of islet endothelial cells to the muscle tissue is of benefit or not needs to be further investigated, but the interaction of human islet endothelial cells to the recipient mouse vasculature is in itself an indication of increased cellular interaction in the presence of MSC. A similar scenario was observed in the *in vivo* setting where CD31 expression in control islets solely could be seen close to the center of the islet mass non-connecting to the recipient vasculature while MSC facilitated the migration of the endothelial cells out into the surrounding tissue where they were interactive with the ingrowing vasculature. Further, MSC was observed at some sites to align with and wrap around the human endothelial cells as to form chimeric blood vessels in a smooth muscle cell behavior. It has been shown *in vitro* that co-cultures of human MSC are stimulated towards a smooth muscle phenotype through the cell-cell interactions with endothelial cells [29]. It has further been reported that islet endothelial cells and recipient blood vessels form chimeric blood vessels in an animal model [30]. Also, in a rat transplantation model in the presence of immunosuppressive drugs the transplanted endothelial cells contributed to chimeric vessels that were functional during 60 days [31]. Here we show the interactions between human MSC, human islet endothelial cells and mouse vasculature. However, the current study lacks the parameters to answer inquiries of enhanced vascular functionality at the chosen site and requires further investigations.

αSMA is normally expressed by vascular smooth muscle cells but can also be expressed by stromal fibroblastic cells in pathological conditions leading to fibrosis [32]. Herein, αSMA expression was seen in the tissue surrounding the graft. Surprisingly, analysis of apoptosis revealed no significant difference between control islets compared to MSC-islets. A possible reason for this could be of a technical matter due to actual loss of apoptotic tissues during sectioning and staining process, as indicated by the increased fibrosis. Without the supportive framework of MSC, the grafted control islets showed accumulation of F4/80 macrophages close to the islet mass, had an active fibroproliferation process that could be detected to a significantly higher extent. However, in the presence of MSC, areas in the islet grafts could be found that were not surrounded by fibrotic tissue. This finding could indicate a healing process of the transplantation area in the presence of MSC.

## Conclusion

The MSC in this model of transplantation contribute to reduced fibrosis, islet endothelial cell migration and interaction with the recipient vasculature and infiltrating macrophages.

## Methods

### Ethics statement

All work involving human tissue was conducted according to the principles expressed in the Declaration of Helsinki and in the European Council’s Convention on Human Rights and Biomedicine. The healthy volunteers donating bone marrow gave written informed consent and the Regional Ethics Committee in Stockholm, Sweden approved the study. Consent for organ donation (for clinical transplantation and for use in research) was obtained verbally from the deceased’s next of kin by the attending physician and documented in the medical records of the deceased in accordance with Swedish law and as approved by the Regional Ethics Committee. The study was approved by the Regional Ethics Committee in Uppsala, Sweden, according to the Act Concerning the Ethical Review of Research Involving Humans. MSC were isolated and expanded from bone marrow of healthy donors as previously described [33] following approval by the ethics committee at Huddinge University Hospital and thereafter cultured and utilized at Uppsala University (EPN Uppsala, Dnr2013/410). All laboratory animal experiments were approved by the local ethics committee (Dnr C261/12, 362/10).

### Isolation and expansion of adult human MSC

The MSC were cultured in MSC medium consisting of Dulbecco’s modified Eagle’s medium-low glucose (DMEM-LG), supplemented with 10 % heat-inactivated fetal bovine serum (FBS from PAA Laboratories GmbH, Pasching, Austria). The release criteria for MSC was based on spindle shaped morphology, cell viability >95 % and flow cytometry of cells with >95 % positivity for CD73, CD90, CD105, HLA-ABC and <5 % for CD14, CD31, CD34, CD45 and HLA-DR as previously described as previously described [33]. The cultures were negative for bacteria, fungi and polymerase chain reaction (PCR)-negative for *Mycoplasma pneumoniae*. In this study, MSC in passages 5–9 from three different donors were used in separate experiments.

### Human islet isolation

Islets of Langerhans were isolated from human pancreas received from brain-dead donors using a semi-automated method [34]. Islet preparations with purity of 69–85 % based upon Ditizone staining were used in separate experiments. Purity of islet preparation was estimated with digital image analysis [35]. Freshly isolated islets and exocrine tissue were cultured free-floating in islet medium, CMRL 1066 culture medium with 10 % ABO serum and supplements, for about 48 h at 37 °C (5 % CO_2_) prior to experiments [36].

### Lentiviral construction and production

A lentiviral plasmid, pBMN (G2L), with the genes encoding *copepod* green fluorescent protein (GFP), codon-optimized *firefly* luciferase separated by a sequence encoding a self-cleaving 2A peptide from *Thosea asigna* virus (T2A) was constructed using pGreenPuro (SBI System Biosciences, Mountain View, CA). A large-scale third generation self-inactivating (SIN) lentivirus batch was produced in HEK-293T cells (Life Technologies, Carlsbad, CA) using polyethyleneimine (Sigma-Aldrich, St Louis, MO) transfection of plasmids pBMN (G2L), pLP1, pLP2 and pVSV-G (Life Technologies) at a ratio of 2:1:1:1. The supernatant was harvested 48 and 72 h post-transfection and concentrated through ultracentrifugation at 75,000 x *g* for 90 min. The viral pellet was resuspended in PBS and stored at −80 °C until further use. The virus titer was determined using the lentivirus qPCR Titer Kit (Applied Biological Materials Inc, Richmond, Canada) following the provider’s instructions.

### Viral transduction of MSC and creation of composite MSC-islets

Viral supernatants (20 μl) were added to 200 000 MSC cultured in 25 cm^2^ flasks in MSC medium supplemented with 8 μg/ml Polybrene (Sigma-Aldrich Inc, Saint Louis, MO, USA). Cells were incubated for 24 h at 37 °C, 5 % CO_2_ and the media was replaced the following day. Transduction efficiency was analyzed for GFP expression using FACScanto II (BD Biosciences, San Diego, CA).

Human islets of Langerhans were manually picked and used one to three days post islet isolation. Islets were incubated with blue cell tracker (1-3 uM Cell Tracker, Molecular Probes, Eugene, OR) in 5cm Sterilin dishes (Sterilin Ltd, New Port, UK) in islet culture medium (see above), 1 h, 37 °C, followed by change of medium and 1h incubation in 37 °C. For creation of composite MSC-islets, we followed earlier established protocols for coating of the islets [13, 37]. In short, approximately 200 islets were added to 5 mL polystyrene tubes (Sarstedt, Numbrecht, Germany) together with 185 000 GFP/luciferase-transduced MSC followed by careful mixing every 30 min during 2 h at room temperature (RT). Islets and composite MSC-islets were thereafter cultured in 5 cm Sterilin dishes with MSC complete medium (see above), 37 °C, overnight.

### Experimental in vivo model

Female NOD-scid ILR2γ^null^ mice were obtained from (MTA TLJ Ref No 005557, Jackson). Within 3–4 days after isolation approximately 200 islets or approximately 200 MSC-islets were upon transplantation dispersed to the abdomen muscle in isoflourane-anesthetized normoglycemic mice with end points and removal of grafts 3 (*n* = 4/group) and 7 (*n* = 5/group) days post transplantation. Islets or MSC-islets were collected and let to sediment in 20−30 ul of islet culture medium using 25G butterfly infusion needles before injection into the muscle tissue. To monitor GFP-luciferase/MSC in the MSC-islet receiving mice, luciferase activity was analyzed using the IVIS-100 Imaging system (Xenogen Corporation, Alameda, CA). An intraperitoneal injection of 10 μl/g body weight D-luciferin (Xenogen) followed by visualization was measured at 5, 10, 15 and 20 min after injection and the mean luciferase signal in each investigated animal was calculated. Luciferase activity measurements were performed each or every second day until endpoint.

### Preparation of tissue for immunohistochemistry

After explantation, mouse abdominal muscles were fixed in 1 % paraformaldehyde (PFA) at 4 °C overnight followed by additional incubation in PBS 4 °C overnight and further in 20 % sucrose/PBS overnight to finally be stored at −70 °C. Longitudinal cryosections (5-7 μm) were cut of the abdominal muscle after mounting in optimal cutting temperature (O.C.T) medium (Tissue-Tech, Sakura Finetek, Zoeterwoude, Netherlands).

### Immunostaining

To enable a complete overview of the graft area within the muscle tissue, longitudinal 5–7 μm cryosections of the grafts were performed. The longitudinal sections were selected through the collected muscle tissue to analyze the tissue transversal (10–20 sections/analyzed marker combination at different section levels within the tissue) and the sections were stained for mouse endothelial cells (CD31 dilution 1:400 (Becton Dickinson (BD) Biosciences, Franklin Lakes, NJ) and human endothelial cells (Alexa-647 conjugated CD31 dilution 1:200, BD Biosciences), human insulin (dilution 1:100, Fitzgerald Industries International, MA), mouse macrophages (F4/80 dilution 1:200, AbD Serotec, Oxford, United Kingdom), fibrotic tissue (Cy5 conjugated α-smooth muscle actin (αSMA) dilution 1:1000, Sigma-Aldrich, St. Louis, MO) [38]. Slides were treated with a blocking/permeabilizing solution; 5 % goat serum/2 % BSA/0,05 % Triton-X, 1 h at RT followed by over night, 4 °C, incubation with primary antibody. Slides were rinsed in TBS-Tween followed by 1 h incubation with secondary antibodies at RT (goat-α-rat Alexa 568, goat-α-rabbit Alexa 405, goat-α-rat Alexa 647, dilution 1:500, Molecular Probes, Eugene, OR, USA). For apoptotic events the ApopTag®Plus In situ Apoptosis Fluorescein Detection Kit (Merck Millipore, Billerica, MA, USA) was applied according to manufacturer’s protocol with addition of staining endocrine tissue using anti-chromogranin A antibody (pre-diluted from DAKO, Glostrup, Denmark) during 1 h at RT. The ethanol/acid fixation step in the ApopTag kit removed the green signal from GFP^+^ MSC thereby avoiding interference with ApopTag fluorescein detection. Slides were rinsed in TBS-Tween followed by 1 h incubation with secondary antibody (donkey-α-rabbit Alexa 568, diluted 1:1000 in 5 % goat serum/2 % BSA, Molecular Probes). Slides were rinsed in TBS followed by mounting with Fluoromount-G (SouthernBiotech, Birmingham, AL) and stored in 4 °C until analyzed.

### In situ staining

Whole muscle were explanted and fixed in 1 % PFA, 4 °C, overnight followed by rinse in TBS and incubation with primary antibodies (mouse CD31 dilution 1:200) diluted in 20 % sucrose/0,05 % Triton-X 4 °C 3–4 days. Secondary antibodies (goat-α-rat Alexa 568 dilution 1:500, Molecular Probes, Eugene, OR) diluted in 20 % sucrose/0,05 % Triton-X were added after TBS-tween rinsing. The muscle were rinsed in TBS and kept in 20 % sucrose until image analysis.

### Image analysis

Microscopy was performed on the cryosections (Zeiss LSM700, Carl Zeiss, Jena, Germany) and *in situ* stained muscle tissue (Zeiss 710 NLO two-photon and confocal microscope (Carl Zeiss) at Science for Life Lab, BioVis Platform, Uppsala). To analyze the quantity of a specific cell marker in a section, each image was analyzed for the amount of events estimated by positive fluorescent staining. Images were split from RGB into red, blue and green single images and made from grey to binary. Percentage of positive events was calculated by the analyze particle function in Fiji image processing software (http://fiji.sc/). Images stained for CD31 were further analyzed for endothelial cell length by using the skeleton plug-in in Fiji (http://fiji.sc/ Analyze Skeleton) and further for distance in relation to islet mass by using the function object neighbors, relation objects and Id primary object, measure correlation and relate objects in the CellProfiler image analysis software (www.cellprofiler.org). CD31 positive structures <40 pixels or 12.5 um were excluded in the analysis to avoid quantification of small dots not representing vascular structures. Also, the analysis of selected CD31 structures for quantification was visually defined as vessel-like structures. Distance analysis of F4/80^+^ cells in correlation to the islet grafts was preformed by measuring distance from all F4/80^+^ events to the closest and second closest islet graft area. Data is presented as mean distance in each analyzed image. Distance analysis was preformed using the functions primary objects, measure object neighbor, measure distance first closest object, measure distance second closest object in the CellProfiler image analysis software. Adobe Photoshop CS6v13.0.2 (Adobe Systems Incorporated, San Jose, CA) was used for all image processing.

### Statistical analysis

Differences of analyzed positive events estimated by fluorescent signal between the groups and time points were compared using the GraphPad Prism (GraphPad Software Incorporated, La Jolla, CA) with significance set to *p* ≤ 0.05 using the non-parametric Mann–Whitney test. Data are presented as mean values with standard deviation (SD).

## Additional files

Additional file 1: Figure S1. Transplantation to the abdomen muscle. (A-C) Images of mouse muscle tissue after injection of control islets and (D-F) MSC-islets. Each graft is marked with an arrow. (TIFF 7989 kb) Additional file 2: Figure S2. IVIS signal of luciferase MSC post transplantation. (A) Three mice (tails labeled with one, two and three lines respectively) analyzed on day 1 post transplantation showing signal localized in dots at distance from the main graft (white arrowheads). (TIFF 4555 kb) Additional file 3: Figure S3. Visualization of longitudinal sectioned islet grafts 7 days post transplantation. (A and B) Control grafts with blue cell tracked islets (blue), accumulating F4/80^+^ cells red in A and purple in B. (C and D) MSC-islet grafts (blue) with MSC (green) and F4/80^+^ cells in purple in C and D. (TIFF 20302 kb) Additional file 4: Figure S4. Apoptosis in the graft tissue three days post transplantation. (A) Apoptosis detected by ApoTaq flourescein (green) in the graft in islet control section (chromogranin A, red). (B) Apoptotic nuclei (green) surrounding the graft (chromogranin A, red) with sparse expression of apoptosis (green) inside the islet mass. (C) Quantification of the apoptotic events at day 3 within the islet graft area showing no differences between the groups. Bars = 100 um. (TIFF 19423 kb)

## Abbreviations

αSMA: alpha smooth muscle actin
CD: Cluster of differentiation
GFP: green fluorescent protein
IBMIR: instant blood mediated inflammatory reaction
MSC: mesenchymal stromal cells
