# Supplementary figures and images for: Mesenchymal stromal cells support endothelial cell interactions in an intramuscular islet transplantation model
Source: Regen Med Res. 2015 Sep 30;3:1. doi: 10.1186/s40340-015-0010-9 (PMC4589952; doi:10.1186/s40340-015-0010-9)

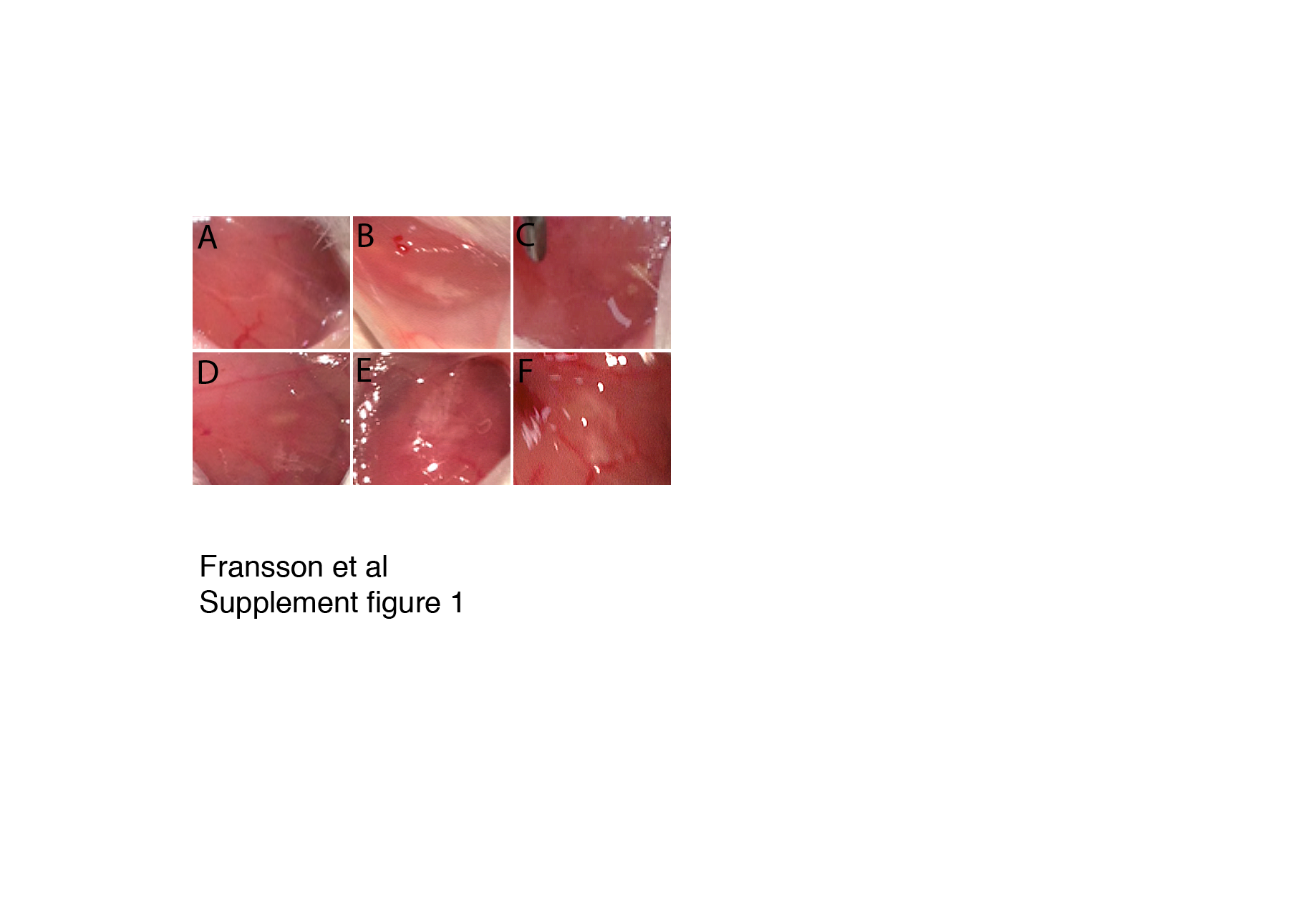

Supplement: Additional file 1: Figure S1. — Transplantation to the abdomen muscle. (A-C) Images of mouse muscle tissue after injection of control islets and (D-F) MSC-islets. Each graft is marked with an arrow. (TIFF 7989 kb) [file 40340_2015_10_MOESM1_ESM.tiff]

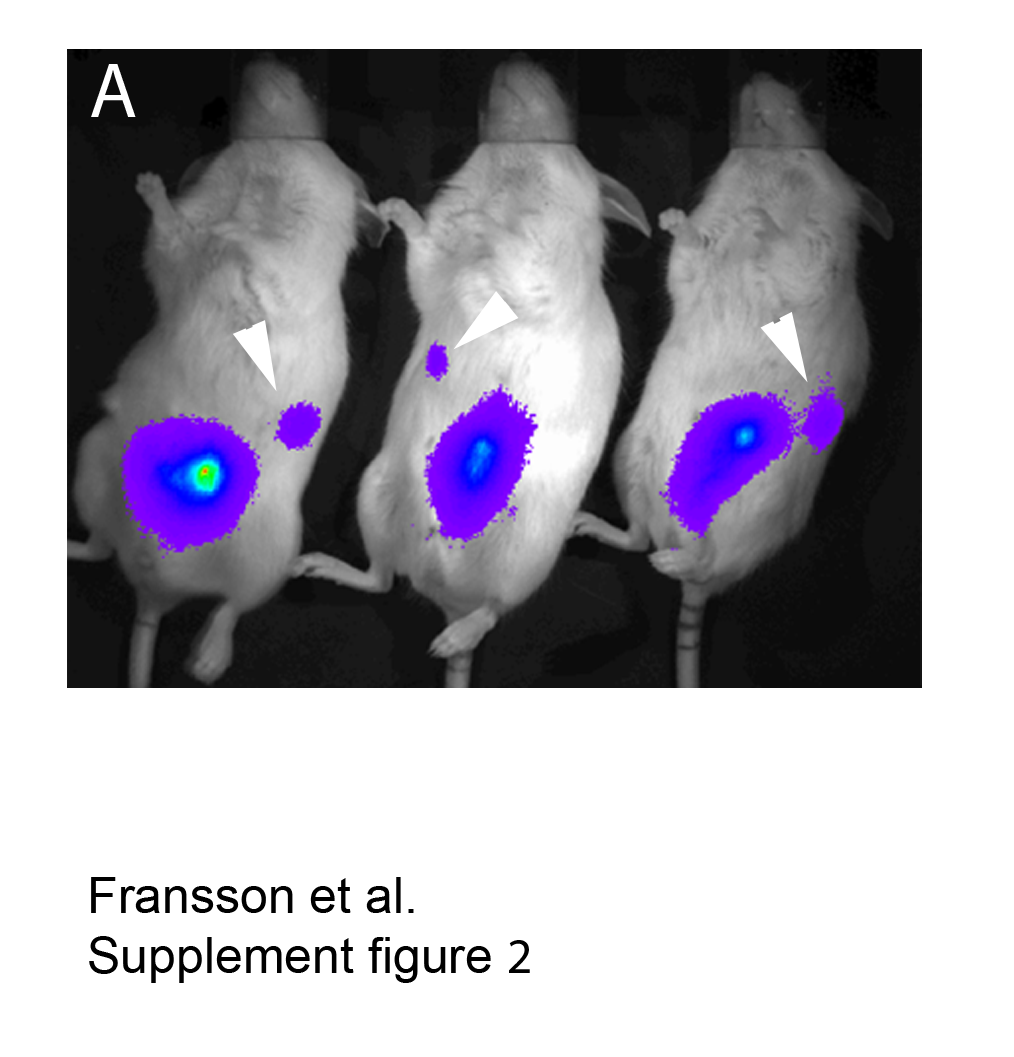

Supplement: Additional file 2: Figure S2. — IVIS signal of luciferase MSC post transplantation. (A) Three mice (tails labeled with one, two and three lines respectively) analyzed on day 1 post transplantation showing signal localized in dots at distance from the main graft (white arrowheads). (TIFF 4555 kb) [file 40340_2015_10_MOESM2_ESM.tiff]

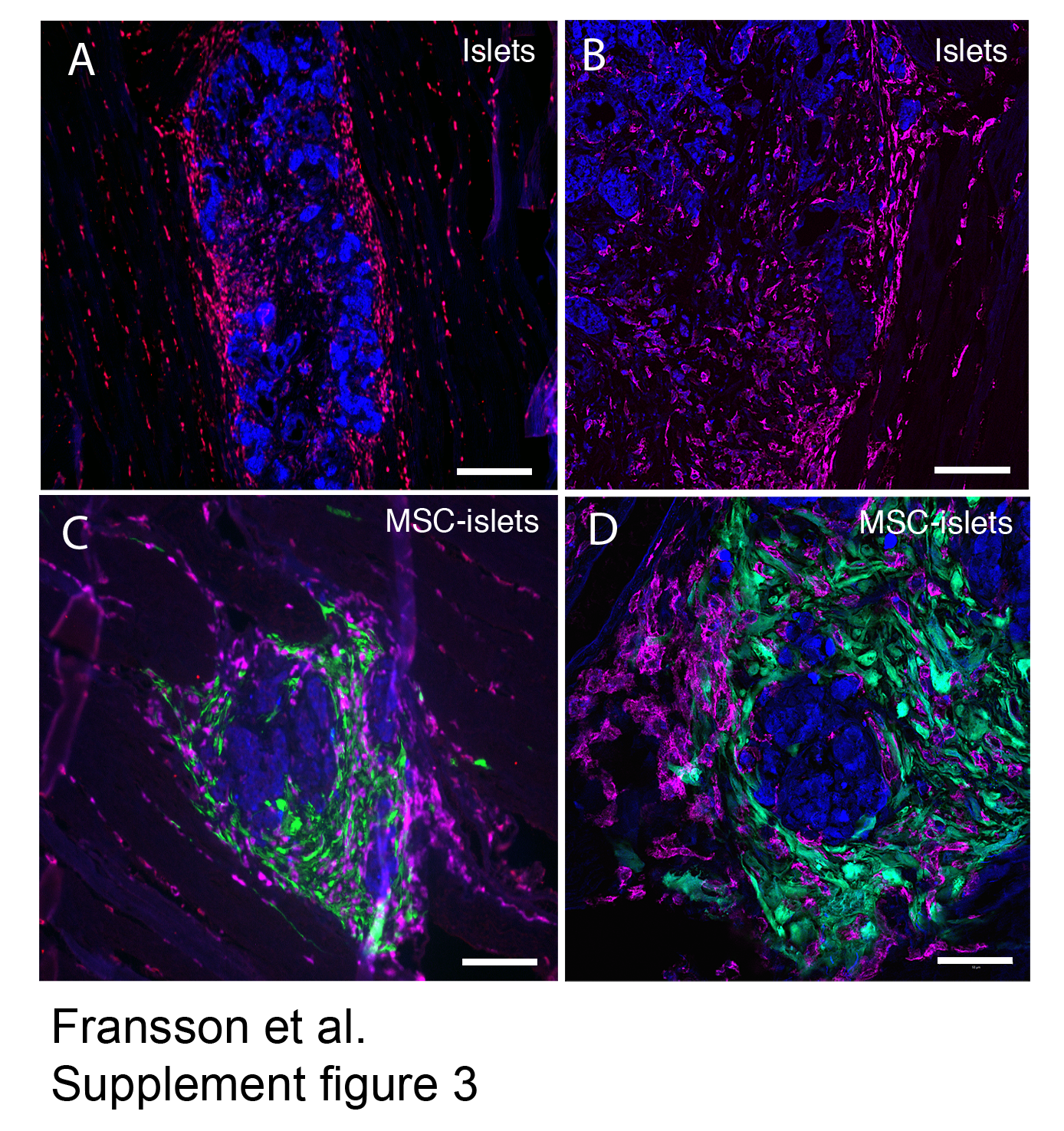

Supplement: Additional file 3: Figure S3. — Visualization of longitudinal sectioned islet grafts 7 days post transplantation. (A and B) Control grafts with blue cell tracked islets (blue), accumulating F4/80+ cells red in A and purple in B. (C and D) MSC-islet grafts (blue) with MSC (green) and F4/80+ cells in purple in C and D. (TIFF 20302 kb) [file 40340_2015_10_MOESM3_ESM.tiff]

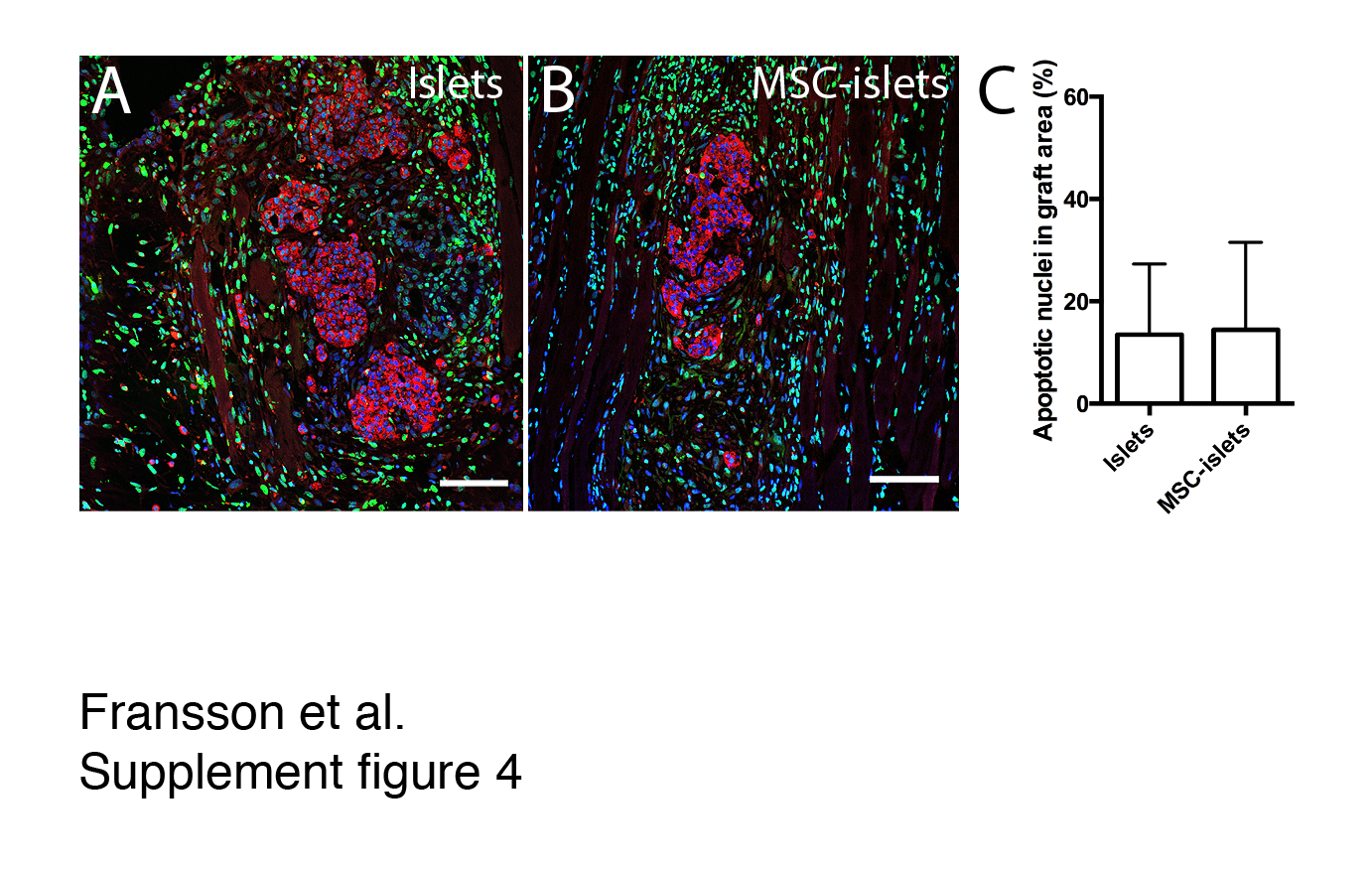

Supplement: Additional file 4: Figure S4. — Apoptosis in the graft tissue three days post transplantation. (A) Apoptosis detected by ApoTaq flourescein (green) in the graft in islet control section (chromogranin A, red). (B) Apoptotic nuclei (green) surrounding the graft (chromogranin A, red) with sparse expression of apoptosis (green) inside the islet mass. (C) Quantification of the apoptotic events at day 3 within the islet graft area showing no differences between the groups. Bars = 100 um. (TIFF 19423 kb) [file 40340_2015_10_MOESM4_ESM.tiff]
